# Editorial: Is two better than one? Exploring tissue microstructure with multi-modal imaging: Quantitative MRI and beyond

**DOI:** 10.3389/fnins.2023.1139400

**Published:** 2023-02-08

**Authors:** Alberto De Luca, Kurt G. Schilling, Andrada Ianus

**Affiliations:** ^1^Image Sciences Institute, University Medical Center Utrecht, Utrecht, Netherlands; ^2^Department of Neurology, University Medical Center Utrecht, Utrecht, Netherlands; ^3^Department of Radiology and Radiological Sciences, Vanderbilt University Medical Center, Nashville, TN, United States; ^4^Vanderbilt University Institute of Imaging Science, Vanderbilt University Medical Center, Nashville, TN, United States; ^5^Champalimaud Research, Champalimaud Foundation, Lisbon, Portugal

**Keywords:** multi-modal imaging, tissue microstructure, quantitative MRI (qMRI), diffusion MRI (dMRI), PET-MR imaging, biophysical tissue models, microscopy, electroencephalography (EEG)

Quantitative MRI methods provide means to characterize tissue structure and function, non-invasively, from microscopic features reflecting tissue architecture to brain wide functional networks. Prolific research studies, developed since the early days of MRI and NMR, have shown that qMRI methods can provide bulk markers of tissue composition (e.g., T1/T2, mean diffusivity, etc.), but also metrics which aim to reflect more specific tissue features, usually derived under various assumptions (e.g., myelin signal fraction, cellular density, microscopic fiber orientation, etc.). Nevertheless, as MRI contrasts are indirect probes of specific physical properties, it is highly likely that no contrast alone can provide a comprehensive evaluation of tissue microstructure. As such, validation and/or integration of MRI with other imaging modalities, and of multiple MRI contrasts, is an important research focus in neuroimaging.

This Research Topic brings together a collection of original research articles that employ multi-modal imaging to enhance the specificity of brain tissue characterization, combining MRI with other imaging modalities, such as electroencephalography (EEG), microscopy, microdissection, and positron emission tomography (PET).

Three of the research articles focus on diffusion MRI (dMRI), a contrast that leverages the diffusion of water molecules in biologic tissues as an indirect probe of tissue microstructure (Novikov et al., 2019). dMRI data can be employed to characterize the tissue composition (i.e., microstructure imaging) as well as to study structural connectivity between different brain regions (i.e., tractography). A challenge in dMRI, both for microstructure imaging and tractography, is to validate the imaging biomarkers and improve the specificity of tissue characterization (Dyrby et al., 2018).

The study of Oliveira et al. integrates *in-vivo* multi-contrast MRI and EEG data to characterize axonal morphology, specifically diameter and g-ratio distribution in the visual transcallosal tract. To achieve this, they develop a joint model that relates the morphological parameters to MRI metrics derived from magnetization transfer and diffusion contrasts, as well as interhemispheric transfer time derived from EEG. This work is an excellent example of joint modeling across imaging modalities to characterize tissue microstructure.

The study of Wu et al. integrated diffusion fiber tractography and postmortem fiber dissection. Doing so, they were able to characterize four major association fiber tracts associated with the language networks of Broca's area. The combined knowledge from these techniques enabled precise descriptions of cortical terminations of these pathways as well as their spatial relationships with one another. Further, they report the existence of a previously undescribed white matter tract, visualized in both tractography and dissections, which may play a role in language function. It is only through multimodal studies that a comprehensive characterization of these connections are possible, which will enable and facilitate studies of their role in brain function.

The study of Radhakrishnan et al. evaluated the correspondence of dMRI measures and cytoarchitectural measures from histology in the mouse brain. They compared measures derived from DTI and NODDI (Zhang et al., 2012) to quantitative cell counts from the Allen reference atlas (Wang et al., 2020). Results showed that DTI and NODDI measures correlated differently with cell counts of specific cell types (e.g., oligodendrocytes, glia, neurons), and that such relations vary on a region specific basis. By combining MRI and histology, this work shows that dMRI measures are sensitive to cytoarchitectural properties, but also highlights the need to test the appropriateness and generalizability of models in a wide variety of cortical regions.

An important step to ease the sharing and analysis of multi-modal data is to establish a well defined structure of the data and metadata that include all necessary information. The article from Bourget et al. extends the Brain Imaging Data Structure (BIDS) for microscopy data (Gorgolewski et al., 2016). First developed for MRI, BIDS is now a well-established specification for sharing and structuring datasets. Extending the data sharing philosophy, Bourget et al., describe methods to support 2D/3D, *ex vivo/in vivo*, micro-CT, and electron and optical imaging microscopy. This was quite a daunting task, as anyone who has worked with microscopy can attest there is little consensus on data specifications, metadata reporting, file formats, nor how to describe varying samples, stains, or fields of view. The BIDS extension for microscopy required several additional entities not typical for MRI, including “sample”, “stain”, and “chunk” features to distinguish samples, stains, and regions. Together, this intuitive specification will facilitate studies described in this issue which aim to explore multimodal or multi-scale imaging, for example combining diffusion MRI with tissue microstructure and histological measures.

The combination of MRI and PET acquisitions is becoming increasingly popular to integrate advanced metabolic information from PET tracers with the multitude of available MR contrasts, facilitated by the advent of commercially available compact PET-MRI systems. Simultaneous PET-MRI (Vandenberghe and Marsden, 2015) is particularly relevant in the study of diseases where PET biomarkers provide specific metabolic information complementing that of MRI markers, such as multiple sclerosis, Alzheimer's disease and brain tumors.

The study by Shan et al. investigates whether performing a functional MRI experiment during PET tracers uptake can impact the results of subsequent metabolic map calculations. This is particularly relevant as previous literature has shown that acoustic, thermal, and electromagnetic effects may confound quantitative tracer uptake curves. On a PET-MRI system, MRI was turned on for the first or second half of the experimental time in a randomized order. Results showed no difference in either mean or voxel-wise plateau values of tracer uptake when comparing between MRI-on and MRI-off periods. However, a considerable increase in the ratio of tracer uptake was detected in the MRI-on period across the whole brain, especially in gray matter, located in sensorimotor, attention, control, default, and auditory networks. Overall, these results suggest the existence of a modest confounding effect of MRI on simultaneous PET scans, and reinforces trust in the possibility to perform advanced studies combining the unique capabilities of these two complementary imaging modalities.

In summary, this Research Topic shows excellent examples of current endeavors to combine MRI with other imaging modalities such as EEG, PET or histology for a better characterization of tissue structure and function across scales.

## Author contributions

AI, AD, and KS contributed to writing this editorial. The project was coordinated by AI. All authors contributed to the article and approved the submitted version.
